# Enumeration of *Mycobacterium leprae* Using Real-Time PCR

**DOI:** 10.1371/journal.pntd.0000328

**Published:** 2008-11-04

**Authors:** Richard W. Truman, P. Kyle Andrews, Naoko Y. Robbins, Linda B. Adams, James L. Krahenbuhl, Thomas P. Gillis

**Affiliations:** Department of Health and Human Services, Health Resources Services Administration, Bureau of Primary Health Care, National Hansen's Disease Programs, Baton Rouge, Louisiana, United States of America; University of Tennessee, United States of America

## Abstract

*Mycobacterium leprae* is not cultivable in axenic media, and direct microscopic enumeration of the bacilli is complex, labor intensive, and suffers from limited sensitivity and specificity. We have developed a real-time PCR assay for quantifying *M. leprae* DNA in biological samples. Primers were identified to amplify a shared region of the multicopy repeat sequence (RLEP) specific to *M. leprae* and tested for sensitivity and specificity in the TaqMan format. The assay was specific for *M. leprae* and able to detect 10 fg of purified *M. leprae* DNA, or approximately 300 bacteria in infected tissues. We used the RLEP TaqMan PCR to assess the short and long-term growth results of *M. leprae* in foot pad tissues obtained from conventional mice, a gene knock-out mouse strain, athymic nude mice, as well as from reticuloendothelial tissues of *M. leprae*–infected nine-banded armadillos. We found excellent correlative results between estimates from RLEP TaqMan PCR and direct microscopic counting (combined r = 0.98). The RLEP TaqMan PCR permitted rapid analysis of batch samples with high reproducibility and is especially valuable for detection of low numbers of bacilli. Molecular enumeration is a rapid, objective and highly reproducible means to estimate the numbers of *M. leprae* in tissues, and application of the technique can facilitate work with this agent in many laboratories.

## Author Summary

*Mycobacterium leprae* is not cultivable in axenic media, and direct microscopic enumeration of the bacilli is complex, labor intensive, and suffers from limited sensitivity and specificity. We describe the use of real-time PCR to provide a rapid, objective and consistent enumeration procedure for *M. leprae*. The procedure is specific for *M. leprae*, has a dynamic range of approximately 6 logs and yields results in only a few hours, including processing time. The procedure was applied to *M. leprae* growing in mouse and armadillo tissues showing excellent correlation with microscopic counting. The benefits of this technique for experimental characterization of leprosy infections and vaccine trials are substantial, and potential applications to clinical specimens could impact patient management by simplifying the assessment of bacterial burden prior to and during drug treatment.

## Introduction

Because *M. leprae* can not be grown on synthetic media, the bacilli must be enumerated by direct microscopic counting. Originally developed by Shepard [1] in the 1960's, this technique has survived as the “gold standard” for enumerating *M. leprae* for almost 50 years. Unfortunately, it is a highly specialized procedure, cumbersome to perform and limited in terms of sensitivity and specificity. Only a few laboratories today have retained the ability to enumerate *M. leprae* using direct microscopy [2],[3].

Various methods have been described to minimize error in direct microscopic counting of *M. leprae*, including the use of special slide coatings, staining procedures, and methods to calibrate microscopes [2],[4]. However, these steps add to the complexity of the technique and the inherent insensitivity of the method requires that multiple samples be processed in large group sizes in order to reduce error. In addition, direct microscopy has limited clinical utility. For example, *M. leprae* cannot be differentiated from other acid-fast bacteria by microscopic examination alone, and clinical assessment of suspect biopsies requires that additional tests also be applied when a mixed infection is suspected [5]–[10].

With the development of nucleic acid-based amplification assays, the identification of difficult to grow microorganisms in tissues, including *M. leprae*, has become routine [11]–[16]. These assays have enhanced our awareness of clinical disease processes, and in some cases have produced new ways to diagnose and monitor mycobacterial infections. Implementing real-time PCR assays adds another potential advantage of direct or indirect quantitation of target DNA. Therefore, we investigated this approach seeking a more precise and reproducible assay for enumerating *M. leprae* in tissues based on the *M. leprae* DNA content of tissue specimens using real-time PCR.

The *M. leprae* chromosome contains a family of dispersed repeats (RLEP) of variable structure and unknown function [17]. Twenty-nine copies of RLEP exist in the chromosome, each containing an invariant 545-bp core flanked in some cases by additional segments ranging from 44 to 100 bps. We identified DNA sequences for TaqMan PCR primers and fluorescent probe from the *M. leprae*-specific, invariant region of RLEP. We tested the specificity of the assay against a number of microorganisms, including cultivable mycobacteria and evaluated the sensitivity of the assay for detecting *M. leprae by* comparing it with direct microscopic counting for accuracy in estimating the number of *M. leprae* under a variety of experimental conditions employing both the mouse foot pad (MFP) model and infected armadillos.

## Materials and Methods

### Bacteria

*M. leprae*, strains Thai-53 or NHDP98 were isolated as previously described [18] and maintained in continuous serial passage in nude mice (Hsd∶Athymic Nude-*Foxn1^nu^*, Harlan Sprague Dawley Inc., Indianapolis, IN). Briefly, *M. leprae* were harvested from nude mouse foot pad tissues after infection for approximately 6 months. Following CO_2_ asphyxiation the hind feet are removed and cleaned with 70% ethanol and Betadine to kill surface contaminants. The skin is removed aseptically and the highly bacilliferous tissue excised, minced and homogenized in 10 ml of Middlebrook 7H12 medium without catalase. Tissue debris is removed by slow speed centrifugation (50×g) for 10 minutes and the bacilli-rich supernatant is pelleted (10 k×g×10 min), resuspended and washed extensively in TE buffer to remove extraneous tissue debris associated with the intact bacilli. The suspension is then enumerated using the method of Shepard et al [1] as described in the MFP Technique below, and viability assessed in axenic culture by the oxidation of ^14^C-palmitate. Viable *M. leprae* obtained through serial passage in nude mice were used to infect other mice and armadillos used in this study [19].

Cultivable mycobacteria were grown to late log phase in Middlebrook 7H9 media plus glycerol, Tween 80 and OADC at appropriate temperatures for optimal growth (Table 1). *M. lepraemurium* was purified from infected mouse spleens and was a gift from I. Brown, Middlesex, England. Genomic DNA was purified from all mycobacteria by enzymatic lysis as described by Belisle and Sonnenberg [20]. Purified genomic DNA from *Streptococcus pyogenes*, *Staphylococcus epidermidis*, *Clostridium perfringens*, *Escherichia coli and Corynebacterium glutamicum* were purchased from American Type Culture Collection (Manassas, VA).

**Table 1:** Specificity of RLEP TaqMan for *Mycobacterium leprae* detection.

Organism	16S rDNA	RLEP	Organisms	16S rDNA	RLEP
*M. leprae*	+	+	*M. marinum*	+	−
*M. avium*	+	−	*M. phlei*	+	−
*M. bovis*	+	−	*M. simiae*	+	−
*M. bovis* BCG	+	−	*M. smegmatis*	+	−
*M. chelonei*	+	−	*M. tuberculosis*	+	−
*M. flavescens*	+	−	*M. ulcerans*	+	−
*M. gordonae*	+	−	*C. perfringens*	+	−
*M. intracellulare*	+	−	*S. epidermidis*	+	−
*M. kansasii*	+	−	*S. pyogenes*	+	−
*M. lepraemurium*	+	−	*E. coli*	+	−
*M. lufu*	+	−			

### Shepard Enumeration and Mouse Foot Pad Technique

Growth of *M. leprae* in the mouse foot pad is determined by direct enumeration of bacilli using the method of Shepard et al [1],[2],[21]. Generally, the bacilli are first inoculated through the planter surface of the foot in 30 ul volumes. A localized infection is established and the bacilli are harvested after a suitable time, often 6 months or more. For enumeration, mice are sacrificed and the plantar surfaces of both hind feet are excised with scalpel and forceps. The tissue is minced with scissors before being transferred to a motorized Potter-Elvehjem tissue grinder where it is homogenized to a fine paste for 1 minute. Trypsin-EDTA (GibcoBRL, Life Technologies, Grand Island, NY) (1 ml) is added and homogenized with the tissue for an additional 30 seconds before the entire preparation is incubated for 15 minutes at 37°C. After incubation the tissue is ground an additional 30 seconds and the entire contents transferred to a glass Mickle homogenizer with 25 glass beads (3 mm), capped, and vibrated for 2 minutes.

For bacterial enumeration, 10 ul of the homogenized liquid is added to 10 ul of calf serum containing 2% phenol. The suspension is mixed thoroughly and spread evenly over three, 1 cm^2^ area circles on a premarked counting slide (Bellco Glass, Inc., Vineland, NJ). After drying in air, slides are fixed in formalin vapor for 3 minutes, using a covered staining dish containing 700 ul of formalin. The fixed slides are then heated on a glass plate over a boiling water bath for 2 minutes. Warmed slides are twice flooded and drained of distilled water containing 0.5% gelatin and 0.5% phenol, and then heated again for 2 minutes between each treatment, and again before being stained. The bacilli are stained using a modified Fite carbol-fuschin for 20 minutes, and decolorized for 30–40 seconds with 5% sulfuric acid in 25% ethanol. Slides are counterstained with crystal violet before a final wash and air drying [1],[4],[21].

Acid-fast bacilli (AFB) are then enumerated by direct examination of 20 oil emersion fields in each of the three, 1 cm^2^ circles, scanning along the horizontal axis of the stained smear using a calibrated microscope. The average number of bacilli in each of three smears is determined and multiplied by the appropriate calibration factor to yield a mean and standard deviation for the AFB count. Care is taken to enumerate only fully stained and intact bacilli avoiding partially stained organisms or those with atypical morphological shapes.

### Samples Enumerated

Three strains of mice were utilized to assess growth and counting efficiency of *M. leprae* using real-time PCR. The strains were 1) fully immunocompetent C57BL/6 mice which permit *M. leprae* growth over an approximate 2-log range of growth from 10^4^ to 10^6^; 2) immune-compromised tumor necrosis factor receptor 1 (TNFR1) knock out (KO) mice (B6.129-Tnfrsf1a^tm1Mak^; The Jackson Laboratory, Bar Harbor, ME). This KO strains exhibits a reduced capacity to control multiplication of *M. leprae*, although not to the extent seen in nude mice, permitting *M. leprae* growth over a 3-log range from 10^4^ to 10^7^; and 3) Athymic nude mice which lack T-cells making them unable to control *M. leprae* infections permitting growth over a 6-log range from 10^4^ to 10^10^.

All studies with animals were previously approved and conducted within the ethical guidelines outlined under the U.S. Public Health Service policy for the care and use of laboratory animals (NHDP IACUC assurance number A3032-01).

#### Conventional Mice

*Vaccine Trial:* C57BL/6 mice (Harlan Inc., Indianapolis, IN) were injected intradermally with 2×10^7^ heat-killed *M. leprae* (n = 20) or normal saline (n = 14). Thirty days later, each mouse was challenged in each hind foot pad with 5,000 viable, nude mouse-derived *M. leprae*. Six months later foot pads were harvested from all mice and *M. leprae* from the infected tissues were prepared for counting as previously described above [1],[21]. The remainder of each bacillary suspension was prepared for TaqMan PCR as described in DNA preparation below.

*Short-term infection:* A fresh suspension of *M. leprae* was harvested from nude mice as described above and serially diluted in HBSS to contain from 1×10^7^–1×10^2^ bacilli in 30 uL. This volume was inoculated through the plantar surface of both hind foot pads (BHFP) of 5 normal BALB/c mice at each dose level. After 4 hours, three mice in each group were sacrificed under CO_2_ and both hind foot pads and popliteal lymph nodes were collected for *M. leprae* enumeration. The remaining two mice in each group were harvested 1 week later and processed in an identical manner.

*Growth of M. leprae in TNF knock out mice:* A total of 24 TNFR1 knock out (KO) mice were inoculated in BHFP through the plantar surface with a 30 uL suspension containing 5000 viable *M. leprae*. The infections were allowed to progress for 6 months when they were harvested for enumeration as described above.

*Growth of M. leprae in nude mice:* Nude mice were inoculated through the plantar surface of both hind foot pads with a suspension containing 1×10^7^ highly viable *M. leprae* in 30 uL of HBSS. The infection was allowed to progress for approximately 6 months and foot pads were harvested when they showed moderate enlargement using the procedure described above. Enumerated samples from 28 mouse harvests were collected and compared in this study.

*Growth of M.leprae in armadillo tissues:* Nine-banded armadillos (*Dasypus novemcinctus*) were inoculated intravenously for large scale propagation of *M leprae* using 1–4×10^9^ highly viable *M. leprae* according to the procedure described before [22]. Animals were allowed to progress through their experimentally induced infections for 18–24 months before they were sacrificed and their livers, spleens and lymph nodes harvested for purification of *M. leprae* [22]. A total of 40 different armadillo liver, spleen and lymph node tissue samples were collected and enumerated in this study.

### Preparation of DNA

#### Mouse-derived *M. leprae*

*M. leprae* DNA was obtained from the tissue homogenates used for microscopic enumeration of acid-fast bacilli. A 200 uL aliquot of the homogenate was subjected to 3 freeze- thaw cycles, mixed with 10 uL of proteinase K (10 mg/ml in buffer, pH 7.5), and incubated at 56 C for 2 hrs.. The samples were then mixed again by vortexing and incubated overnight. The proteinase K was inactivated by heating to 95°C for 1 hour and an additional 40 uL of TE was added to bring the volume to 250 uL. Samples were then mixed and diluted 1∶4 prior to testing.

#### Armadillo-derived *M. leprae*

A 1.0 gm sample of highly bacilliferous armadillo liver, spleen or lymph node was homogenized in a motorized Potter-Elvehjem tissue grinder containing 4 mL of 7H12 broth and frozen at −70 C. Upon thawing the homogenate was diluted 1∶100 in DH_2_0 and processed with DNeasy (Qiagen, Inc., Valencia, CA) according to the manufacturers recommendations. Briefly, 10 µL of the 1∶100 tissue homogenate was added to 80 µL of ATL buffer with 20 µL of proteinase K solution. The sample was mixed and incubated for approximately 1 hour at 56° C with occasional mixing. After incubation samples were mixed thoroughly and 200 µL of AL buffer and 200 µL of ethanol were added before mixing again. This mixture was transferred to a spin column and centrifuged at 6000×g for 1 min discarding the flow through volume and transferred to a new catch tube. 500 µL of AW1 buffer was added and centrifuged at 6000×g for 1.0 min. The flow through was again discarded and the column transferred to a new catch tube. 500 µL of AW2 buffer is then added and centrifuged at 20,000×g for 3.0 min discarding the flow through and transferring the column to a sterile 1.5 mL tube. 200 µL of the elution buffer was added and the column is centrifuged at 6000×g for 1 min. The eluate was collected and diluted 1∶4 prior to testing.

### TaqMan Assays

Primers and probe for the RLEP TaqMan PCR were selected from a common region of the RLEP family of dispersed repeats. *M. leprae* RLEP DNA sequences were acquired from the Sanger Center (www.sanger.ac.uk) and aligned for regions of identity using Omiga 2.0 software (Oxford Molecular Ltd., Madison, WI). RLEP primers and fluorescent probe were chosen using Primer Express software (PE Applied Biosystems, Foster City, CA) based on criteria established for TaqMan PCR reactions. All reagents used in the TaqMan assay were recommended by the manufacturer (PE Applied Biosystems), including AmpErase UNG enzyme and AmpliTaq Gold DNA polymerase. PCR cycling conditions were 40 cycles with 60°C annealing/extension temperature for 60 seconds and 95°C denaturating temperature for 15 seconds. PCR and data analyses were performed on a 7300 RealTime PCR System (Applied Biosystems, Foster City, CA).

## Results

### Primers, Sensitivity and Specificity

RLEP TaqMan primers and probe were selected by aligning DNA sequences from RLEP 1, 2, 3 and 4. A region of RLEP was selected in which the four families of dispersed repeats were identical and analyzed for optimal TaqMan primers and probe. The sequence selected was 5′**-GCAGTATCGTGTTAGTGAA**CAGTGCA*tcgatgatccggccgtcggcg*GCA**CATACGGCAACCTTCTAGCG-3′**. Capital letters in bold represent the sequence on which the forward and reverse primers were built. The sequence in lower case italics was selected for building the fluorescent TaqMan probe. When forward and reverse primer sequences were blasted against the *M. leprae* genome, 19 regions were identified with identical sequences. Another 8 regions were identified with high homology to the primers but amplification would not be likely because of 3-prime mismatches with these primers. Accordingly, amplification of a single *M. leprae* chromosome with these primers should result in 19 copies of RLEP.

**Figure 1:**
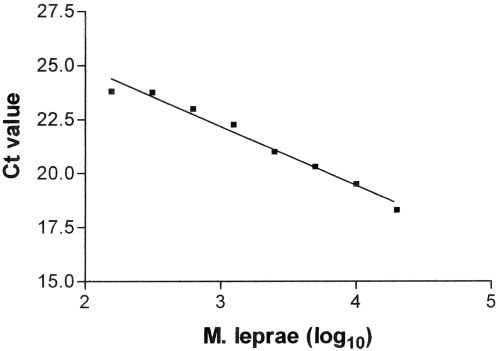
RLEP TaqMan PCR results from titration of nude mouse-derived *M. leprae*. Serial 2-fold dilutions of *M. leprae* were made from 2×10^6^ to 1.56×10^4^/ml. Ten microliters of each dilution were tested in triplicate representing 2×10^4^ to 156 *M. leprae* in the test sample, respectively. The ordinate is PCR cycle number at threshold and the abscissa is number of *M. leprae* (log_10_). Standard deviations did not exceed 0.5% of mean at any dilution.

Sensitivity of the RLEP TaqMan PCR assay was tested with both purified *M. leprae* DNA and nude mouse-derived *M. leprae*. A titration of *M. leprae* DNA in the TaqMan PCR using RLEP primers/probe gave a lower limit of detection of 10 fg equaling approximately 3 organisms based on the *M. leprae* chromosome of approximately 3.27 Mb (data not shown). While these conditions measure the sensitivity of the assay under ideal circumstances (no inhibitors), a more realistic assessment of the detection limit was determined using *M. leprae* harvested from infected mouse tissues. Using nude mouse-derived *M. leprae* as a source of DNA the RLEP TaqMan PCR was able to detect approximately 300 *M. leprae* (Fig. 1).

Specificity of the RLEP TaqMan PCR for *M. leprae* DNA was determined by testing purified genomic DNA from 16 mycobacterial species, 10 of which are associated with human diseases, three gram positive microorganisms often associated with skin infections and *E. coli* (Table 1). In order to monitor genomic DNA for efficient amplification by PCR, samples were tested for reactivity in a separate PCR designed to detect 16S rDNA [23]. All samples tested for 16S rDNA gave a strong signal based on agarose gel electrophoresis when amplifying 10 pg of genomic DNA for 35 cycles. In contrast, RLEP TaqMan PCR was positive only for *M. leprae* DNA when samples were tested at the same concentration using 40 cycles.

### Correlation between Molecular Enumeration with RLEP and Direct Counting

**Figure 2:**
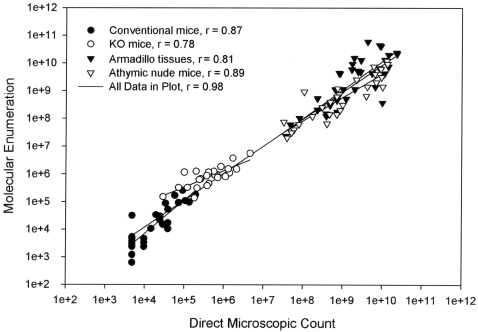
Comparison of direct microscopic counting of AFB per standard volume with enumeration of *M. leprae* by RLEP TaqMan PCR from tissues originating from a variety of host animals. Symbols identify individual samples from sets of conventional, TNFR1 knock-out (KO), and congentially athymic nude mice, as well as from nine-banded armadillos. Pearson's coefficient (r^2^) is calculated for each tissue set. Enumeration estimates for all tissues combined showed high correlation (r^2^ = 0.96) between the “gold standard' direct microscopic counting and estimates based on RLEP TaqMan PCR.

After enumeration by direct microscopic counting, we extracted DNA for enumeration by RLEP TaqMan PCR using the highest enumerated sample of each tissue type to establish a standard curve for those tissues. As shown in Figure 2, direct microscopic counts ranged between 4.8×10^3^ and 2.3×10^10^ bacilli. Estimates based on RLEP TaqMan PCR ranged from 623 organisms in conventional mouse foot pad tissues, to 5.8×10^10^ bacilli in each gram of armadillo tissue. For Molecular Enumeration, Coefficient of Variation (CV) between individual replicates averaged 14.03% (Mode 0.53%, Median 5.58%). Similar CV data was not available for the direct microscopic counts and no values were excluded based on CV. Enumeration estimates based on RLEP showed good correlation with direct microscopic counting with coefficients (Pearson's r) ranging from 0.78 to 0.89 for individual tissue types examined. Best results were seen with tissue sets that had a broad range of estimated bacillary counts. No significant difference in counting efficiency was seen between the various types (liver, spleen or lymph node) of armadillo tissues examined (data not shown). In combination across all tissues examined, RLEP showed a correlation of 0.98 (Pearson's) with direct microscopic counting.

### Application of RLEP TaqMan PCR assay in experimental procedures

#### Fate of *M. leprae* in short-term infections

To better understand the fate of *M. leprae* after inoculation into the mouse, we used the RLEP TaqMan PCR to enumerate the number of bacilli remaining in the foot pad 4 hours and 1 week after inoculation. The foot pads were injected with equal volumes containing concentrations of bacilli ranging from 1×10^7^ to 1×10^2^ *M. leprae* per foot pad (Table 2). More *M. leprae* was retained in the first few hours after inoculation than 1 week later and inocula containing higher concentrations of *M. leprae* demonstrated better retention (ranging from 2.84% to 20.8%). *M. leprae* inoculated at doses lower than 1×10^5^/ foot pad did not yield detectable results (data not shown).

**Table 2:** The number and percent of bacilli recovered from conventional mouse foot pads within 4 hours and 1 week post inoculation with varying doses of *M. leprae* as measured by RLEP PCR.

4 hr			% of Dose Retained	1 wk		% of Dose Retained
Dose Bacilli Given	Site	Bacilli/Foot Pad Recovered		Site	Bacilli/Foot Pad Recovered	
1.00E+07	LF1	2.08E+06	20.80%	LF4	1.25E+05	1.25%
	RF1	1.48E+06	14.80%	RF4	2.70E+05	2.70%
	LF2	1.15E+06	11.50%	LF5	3.20E+05	3.20%
	RF2	8.67E+05	8.67%	RF5	7.15E+05	7.15%
	LF3	5.61E+05	5.61%			
	RT3	1.74E+06	17.40%			
1.00E+06	LF1	1.23E+05	12.28%	LF4	6.14E+04	6.14%
	RF1	8.27E+04	8.27%	RF4	4.95E+04	4.95%
	LF2	5.07E+04	5.10%	LF5	6.68E+03	0.67%
	RF2	8.39E+04	8.39%	RF5	7.80E+04	7.80%
	LF3	2.94E+04	2.94%			
	RF3	3.73E+04	3.73%			
1.00E+05	LF1	NR	NR	LF4	2.13E+03	2.10%
	RF1	NR	NR	RF4	2.81E+03	2.80%
	LF2	6.65E+03	6.65%	LF5	4.31E+03	4.31%
	RF2	2.87E+03	2.87%	RF5	7.39E+03	7.39%
	LF3	2.84E+03	2.84%			
	RF3	2.86E+03	2.86%			
Average (SD)			**8.42% (5.57%)**			**4.21% (2.47%)**

LF = Left Foot with dose animal number, RF = Right Foot with dose animal number, NR = Not Run. SD = Standard Deviation of average percent bacilli retained in the foot pad.

On average, 4 hours after inoculation into the foot pad only 8.42% (+/−5.57%) of any inoculum could still be detected within the foot. After 1 week, the degree of individual variation in the number of bacilli retained within the foot was markedly decreased for all inoculum dose levels; but there appeared to be some continued loss of bacilli from the site. On average, for all the inoculum dose levels considered, only 4.21% (+/−2.47%) of the bacilli originally injected into the mouse foot pad could be detected there after 1 week of incubation.

In an attempt to account for bacilli draining from the foot pad we also examined the popliteal lymph node associated with each foot. Within the first 4 hours these lymph nodes were uniformly small and unperturbed. After 1 week they appeared noticeably enlarged, however, *M. leprae* could not be enumerated in these nodes using RLEP TaqMan at either time period. If the bacilli are retained by these nodes, the amount of amplifiable DNA was below the detectable level of our assay.

#### Estimating Vaccine Efficacy

**Figure 3:**
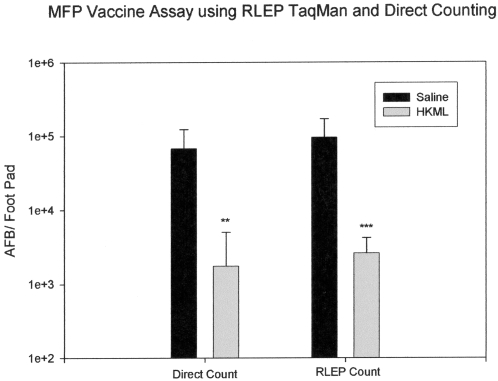
A comparison of RLEP TaqMan PCR and *M. leprae* counting results from a vaccine trial using conventional C57/B mice. Bars represent mean plus the standard deviation for each group. ** = probability of statistical significance (p)<0.01, and *** = probability of statistical significance (p)<0.001.

To determine the effect of host resistance towards *M. leprae* on the efficiency of molecular enumeration, we compared counting results obtained with the two techniques in a standard mouse foot pad (MFP) vaccine study. The seminal work of Shepard, et al [24] and a large number of subsequent studies have demonstrated the suppressive effect of potent vaccines, such as BCG and heat-killed *M. leprae*, on the growth of *M. leprae* in the mouse foot pad model. Briefly, prior sensitization with heat-killed *M. leprae* will result in 1 to 2 logs growth suppression in the mouse foot pad. In our studies mice were vaccinated with heat-killed *M. leprae* (HKML) or saline, as a sham vaccine, and challenged with 5000 viable *M. leprae* in their foot pads thirty days following vaccination. *M. leprae* enumerations were performed 6 months after challenge by RLEP TaqMan PCR and direct microscopic counting. The results of the vaccine trial are shown in Figure 3.

Vaccination with HKML resulted in a significant reduction in the growth of *M. leprae* in the foot pads (p<0.01, Kruskall-Wallace and Dunn's Multiple Comparison's Test) that was detectable by both enumeration techniques. Bacillary counts below 10^3^ are generally not detectable by direct microscopy, but with RLEP enumeration, numerical estimates were derived for 11 samples that otherwise were at or below the threshold of detection for direct microscopic counting. An effective host response that successfully limited growth of the bacilli in the foot pad did not adversely influence the ability to enumerate organisms based on amplification of RLEP DNA amplification.

## Discussion

These results demonstrate that a simple, reproducible test based on genomic DNA can be used to quantify *M. leprae* in infected tissues. The real time PCR assay yields results similar to those obtained from conventional direct microscopic counting methods, is highly specific, sensitive, and is easily adapted to large scale batch processing of samples. Molecular quantification of *M. leprae* based on amplification of RLEP TaqMan PCR is a suitable replacement for direct microscopic counting of bacilli.

The quantitative sensitivity of RLEP PCR is within the range of other PCR detection assays for *M. leprae* DNA developed based on a single-copy gene [9]. A major difference between the two assay systems, however, is the time required for analytical testing, and the ability to quantify multiple batch samples at all time points during thermocycling. For example, the 18-kDa traditional PCR with specific probe hybridization, which we developed earlier, requires approximately 48 hours to complete, whereas the RLEP TaqMan PCR can be accomplished with full analysis in as little as 6 hours. Conventional direct microscopic enumeration requires several hours per sample and has no time savings associated with batch processing.

The greater sensitivity of the RLEP TaqMan PCR can be especially useful for comparative growth studies in the MFP model and some *in vitro* techniques. The threshold limit of detection for direct microscopic counting is approximately 1×10^4^ bacilli. Growth results below those levels are not reliable and data baselines in MFP studies are usually plotted as 1×10^4^ or erroneously coded as zero. Since the upper level of growth in the conventional mouse foot pad plateaus at around 1×10^6^ bacilli for BALB/c mice, and perhaps even lower for some other mouse strains, statistical significance in MFP growth results must be drawn from within only a narrow 2 log window.

RLEP TaqMan PCR yields reliable quantitative growth results with less variation at a lower detection threshold than direct microscopic counting (about 300 organisms) and the counting efficiency is not influenced by cellular immune processes. The greater sensitivity of RLEP TaqMan PCR can benefit discernment of statistically significant results within more narrow ranges. In addition, since *M. leprae* is also a notoriously slow growing organism, more sensitive enumeration methods also could lead to shortening MFP trials which now often require 7–12 months to reach completion.

Most of our knowledge about the microbiological characteristics of *M. leprae* is derived from mouse foot pad studies. In the classic Shepard model, mice are typically inoculated in the foot pad with between 5000–10000 bacilli, and the growth of these organisms is assessed after 120–360 days. Even though a large bolus is deposited into the foot, Levy and others observed that the number of bacilli retained in the foot pad 1 week after inoculation was too low to visualize with direct microscopy [25],[26]. The fate of these organisms remains unknown, but our observations that some 90% of the bacilli are lost from the foot within only a few hours after inoculation is in keeping with those original results and confirms a more immediate time for their loss.

Foot pad inoculation was originally developed as a means to provide *M. leprae* a low temperature growth environment. However, the architecture of the foot pad is not ideal for retention of an inoculum or for supporting the growth of obligate intracellular organisms, such as *M. leprae*. The soft tissue of the foot pad contains few phagocytic cells and consists mainly of dermal and epidermal cells, along with striated muscle. While *M. leprae* can invade striated muscle cells and other non-professional phagocytes [27], their preferred host cell is the macrophage, and sustained local growth of *M. leprae* in the foot pad requires a continuous influx of new macrophages to the site.

It is notable that popliteal lymph nodes of the mice studied here showed enlargement within one week of foot pad inoculation, even in absence of detectable bacilli in those nodes. The specific mechanisms potentially involved in recruiting macrophages to the foot pad are well beyond the scope of this paper; however, these observations support the notion that there is some systemic stimulation following inoculation of the foot pad and these processes may play an important role in establishing and maintaining that localized infection.

Although MFP is the oldest and most widely used method to propagate *M. leprae*, there is much that remains unknown about the technique. Methods that might enhance the growth environment for *M. leprae* in the foot pad by priming the host beforehand, or pre-populating the foot pads with receptive macrophages could benefit our ability to better exploit this model. Regardless, evolution of more sensitive methods to detect *M. leprae* in tissues, such as RLEP TaqMan PCR, can aid that development and help advance this reliable model.

Other gene targets also can likely be used for relative quantification of *M. leprae*. Our results with the RLEP TaqMan PCR are in keeping with those reported earlier for quantification of *M. leprae* based on genetic sequences in the proline-rich antigen region that used purified DNA as a comparative standard [28]. However, the accuracy of estimates based on comparison to purified DNA standard depends entirely on the efficiency of DNA isolation from different tissues, and the inter-run reproducibility of the extraction method. The use of pre-enumerated standards as employed here (and also available from the NIAID Leprosy Research Support Contract), can help eliminate the inaccuracy inherent in variable recovery of DNA in different runs or conditions, and permits ready comparison of results between individual laboratories.

Molecular enumeration of *M. leprae* using the RLEP TaqMan PCR is a rapid and more accurate method to quantify *M. leprae* in tissues that can have wide applicability in research. The DNA based technique is more sensitive and reproducible than direct microscopic counting, requires less technical expertise, and can permit ready comparisons of results between laboratories. Utilization of this or other molecular based techniques to enumerate *M. leprae* will likely aide more careful investigation of growth results in a variety of model systems, and will enhance our ability to propagate this and other difficult to grow microorganisms.
